# Nonsense-Mediated mRNA Decay as a Mediator of Tumorigenesis

**DOI:** 10.3390/genes14020357

**Published:** 2023-01-30

**Authors:** Preeti Nagar, Md Rafikul Islam, Mohammad Alinoor Rahman

**Affiliations:** 1 Department of Biochemistry and Molecular Biology, University of Arkansas for Medical Sciences, Little Rock, AR 72205, USA; 2 Winthrop P. Rockefeller Cancer Institute, University of Arkansas for Medical Sciences, Little Rock, AR 72205, USA

**Keywords:** nonsense-mediated mRNA decay, splicing, gene expression, cancer

## Abstract

Nonsense-mediated mRNA decay (NMD) is an evolutionarily conserved and well-characterized biological mechanism that ensures the fidelity and regulation of gene expression. Initially, NMD was described as a cellular surveillance or quality control process to promote selective recognition and rapid degradation of erroneous transcripts harboring a premature translation-termination codon (PTC). As estimated, one-third of mutated and disease-causing mRNAs were reported to be targeted and degraded by NMD, suggesting the significance of this intricate mechanism in maintaining cellular integrity. It was later revealed that NMD also elicits down-regulation of many endogenous mRNAs without mutations (~10% of the human transcriptome). Therefore, NMD modulates gene expression to evade the generation of aberrant truncated proteins with detrimental functions, compromised activities, or dominant-negative effects, as well as by controlling the abundance of endogenous mRNAs. By regulating gene expression, NMD promotes diverse biological functions during development and differentiation, and facilitates cellular responses to adaptation, physiological changes, stresses, environmental insults, etc. Mutations or alterations (such as abnormal expression, degradation, post-translational modification, etc.) that impair the function or expression of proteins associated with the NMD pathway can be deleterious to cells and may cause pathological consequences, as implicated in developmental and intellectual disabilities, genetic defects, and cancer. Growing evidence in past decades has highlighted NMD as a critical driver of tumorigenesis. Advances in sequencing technologies provided the opportunity to identify many NMD substrate mRNAs in tumor samples compared to matched normal tissues. Interestingly, many of these changes are tumor-specific and are often fine-tuned in a tumor-specific manner, suggesting the complex regulation of NMD in cancer. Tumor cells differentially exploit NMD for survival benefits. Some tumors promote NMD to degrade a subset of mRNAs, such as those encoding tumor suppressors, stress response proteins, signaling proteins, RNA binding proteins, splicing factors, and immunogenic neoantigens. In contrast, some tumors suppress NMD to facilitate the expression of oncoproteins or other proteins beneficial for tumor growth and progression. In this review, we discuss how NMD is regulated as a critical mediator of oncogenesis to promote the development and progression of tumor cells. Understanding how NMD affects tumorigenesis differentially will pave the way for the development of more effective and less toxic, targeted therapeutic opportunities in the era of personalized medicine.

## 1. Introduction

**Figure 1:**
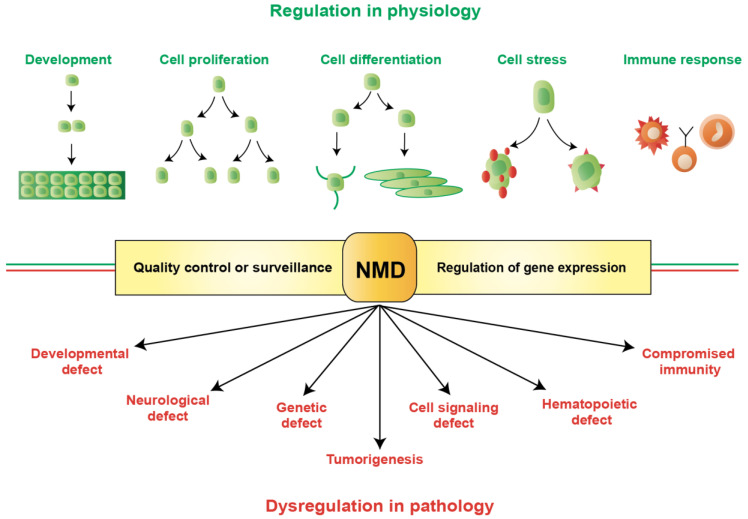
Regulation and dysregulation of nonsense-mediated mRNA decay (NMD) in physiology and pathology. NMD is an evolutionarily conserved pathway comprising a dual function to modulate gene expression: a surveillance or quality control mechanism to recognize and degrade erroneous mRNAs selectively; and a regulatory mechanism to control transcript abundance. By regulating gene expression, NMD promotes diverse physiological functions (shown at the top), including development, cell proliferation, differentiation, cellular stress, immune responses, etc. Mutations or alterations that impair the function or expression of proteins associated with the NMD pathway can be deleterious to cells and may cause pathological consequences (shown in the bottom), as implicated in developmental and neurological disability, genetic defect, cell signaling defect, hematopoietic defect, compromised immunity, and cancer. Therefore, NMD is strictly regulated in cells to safeguard the fidelity of gene expression.

Eukaryotic cells have a sophisticated and strictly synchronized gene expression to promote diverse physiological functions, using a limited number of genes, and to maintain genome integrity in response to various environmental challenges. This highly regulated gene expression comprises a complex series of intersected mechanisms among which messenger RNAs (mRNAs) play pivotal roles. To ensure the fidelity of gene expression, mRNAs are strictly inspected through several quality control mechanisms to identify potential errors that may produce defective proteins with compromised functions or deleterious proteins with dominant negative effects. Nonsense-mediated mRNA decay (NMD) is one such mechanism, which was initially described as an evolutionarily conserved surveillance mechanism to selectively degrade erroneous transcripts [1,2]. Transcripts with a premature translation-termination codon (PTC) are recognized and processed by NMD for cleavage and elimination. PTCs generally arise from genetic mutations (such as nonsense or frame-shift mutations), genomic rearrangements, errors in RNA splicing, regulated alternative splicing, and alternative translation initiations [3,4,5]. In addition to surveillance, NMD also regulates the abundance of a large repertoire of physiological transcripts (approximately 10% of the human transcriptome) [6,7]. Therefore, NMD plays a crucial role in gene expression that shapes the transcriptome to support cellular functions. For example, NMD has been implicated in several physiological processes, including development, cell proliferation, differentiation, cellular stress, and immune responses (Figure 1) [8,9]. Since NMD is a central mechanism in the gene expression pathway, this can affect different biological processes by affecting gene functions; therefore, it is a crucial target for cellular vulnerabilities. Indeed, NMD has been linked to a variety of human pathologies, including neurological disorders, genetic defects, developmental abnormalities, compromised immunity, hematopoietic defects, and cancer (Figure 1) [1,2,10,11].

In this review, we concisely discuss different modes of regulation of NMD as a mediator of tumorigenesis to extend our understanding of the field. Based on accumulating knowledge, we systematically describe how tumor cells exploit NMD for their survival benefits, with extensive mechanistic insights. This will broaden our fundamental understanding of this intricate pathway in cancer cells versus normal cells and may provide critical information for targeting the NMD pathway for cancer therapy.

## 2. Physiological Mechanisms of NMD

NMD is a translation-dependent process, which is promoted by the dynamic interactions between mRNA and a series of NMD-associated proteins, and upon a complex choreography of events between mRNA-bound proteins. The NMD pathway comprises several complicated regulatory events consisting of the discrimination of NMD substrates from other mRNAs, activation of NMD, and mRNA degradation. The molecular mechanisms associated with the NMD pathway are still emerging. To date, several different NMD pathways have been documented (such as the exon junction complex (EJC)-dependent and the EJC-independent pathways, see below), differing in various fundamental aspects that enhanced the visibility of this complex pathway to better explain its role in physiology and pathology.

**Figure 2:**
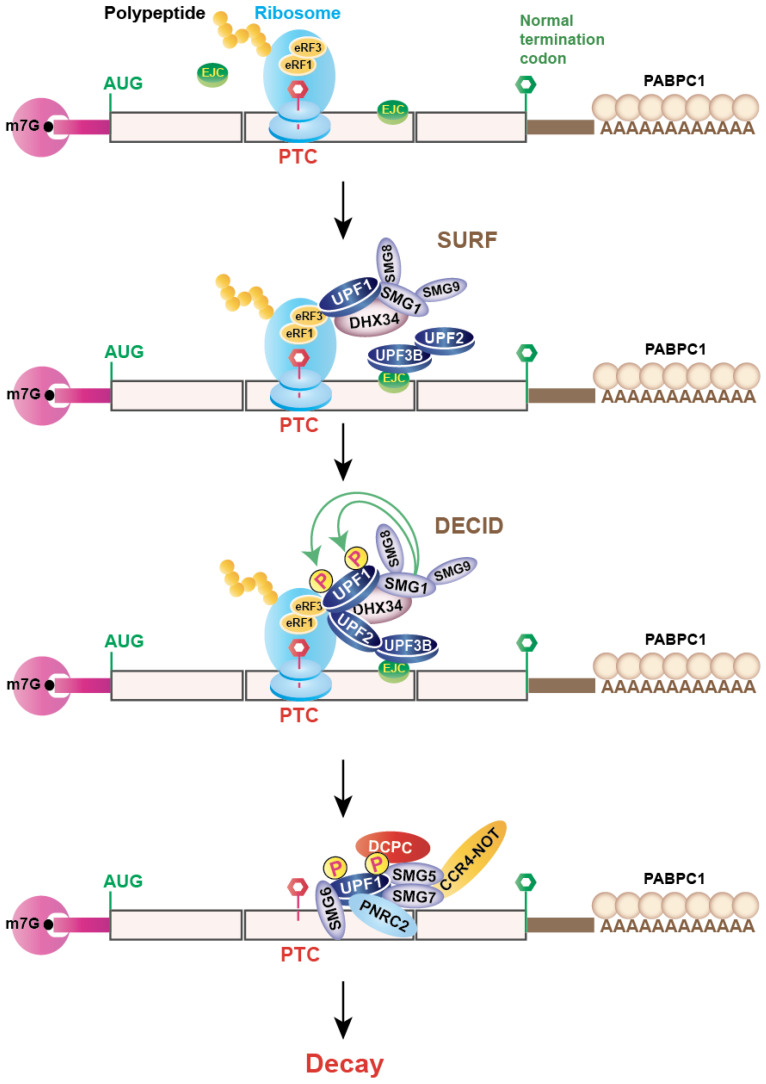
Schematics of the nonsense-mediated mRNA decay (NMD) pathway in mammalian cells. When a translating ribosome encounters a premature termination codon (PTC), the core NMD factor UPF1 interacts with the eukaryotic release factors eRF1 and eRF3, bound to the terminating ribosome, and induces premature translation termination. After this, the SURF complex is formed with SMG1 associated with SMG8 and SMG9, UPF1, eRF1, and eRF3. The SURF complex is then transformed into a decay-inducing complex (DECID), where UPF1 interacts with UPF2-UPF3B, either bound to the downstream EJC (the EJC-dependent NMD model) or disseminated in the cytoplasm (the EJC-independent NMD model). Subsequently, SMG1 induces the phosphorylation of UPF1. At this stage, translation is terminated with the separation of the ribosomal subunits, release factors, and developing peptide. Phosphorylated UPF1 triggers the mRNA decay by promoting the recruitment of different mRNA decay factors: SMG6 causes an endonucleolytic cleavage; SMG5-SMG7 heterodimer recruits the CCR4-NOT deadenylation complex, and/or PNRC2, and subsequently recruits the decapping complex (DCPC) to remove the cap-binding complex and the poly(A) tail. This facilitates 5′-to-3′ and 3′-to-5′ RNA degradation by XRN1 and the RNA exosome, respectively.

The way by which NMD discriminates its targets is still not entirely clear. Currently, two major mechanisms are proposed in the field to explain how cells select the mRNA target: the EJC-dependent model (Figure 2) and the EJC-independent model [12]. EJC is a key regulatory player in the NMD pathway. EJC includes a core (comprising eIF4AIII, MAGOH/MAGOHB, and Y14) and a peripheral shell (consisting of more than a dozen proteins, including NMD factors) [13,14,15,16]. It is deposited ~20–24 nucleotides upstream of most (~80%) exon–exon junctions in a sequence-independent manner during the last step of pre-mRNA splicing [17,18]. EJC provides a functional bridging to facilitate metabolism between nuclear and cytoplasmic mRNA and helps to recruit different factors essential for mRNA export, translation, and NMD. During the first round of translation (pioneer round of translation), EJCs are dissociated from the mRNA by translating ribosomes [13,14,15,16,19,20]. In certain transcripts that contain PTC > 55 nucleotides upstream of an exon–exon junction, the ribosome stops at PTC and cannot displace the EJCs from the transcript downstream of the PTC [13,21]. This allows the interactions between EJC and NMD factors to trigger NMD [13,22]. At this stage, several NMD-associated factors come into play. The most important core NMD factor is up-frameshift protein 1 (UPF1), an RNA helicase, which interacts with the eukaryotic release factors eRF1 and eRF3, bound to the terminating ribosome, and induces premature translation termination. After this, the SURF complex is formed with SMG1 associated with SMG8 and SMG9, UPF1, eRF1, and eRF3, which is facilitated by the RNA helicase DEAH box polypeptide (DHX34) [23]. The SURF complex is then transformed into a decay-inducing complex (DECID) in which UPF1 interacts with UPF2-UPF3B, either bound to the downstream EJC (EJC-dependent NMD model) or diffused in the cytoplasm (EJC-independent NMD model) [12,24,25]. Subsequently, SMG1 induces the phosphorylation of UPF1 [24,26]. At this moment, translation is terminated with the separation of the ribosomal subunits, release factors, and developing peptide. Phosphorylated UPF1 triggers mRNA decay by two routes, depending on the involvement of different NMD factors and the direction of degradation. One route is SMG6-mediated, and the other is SMG5-SMG7 heterodimer or SMG5 and PNRC2 (Proline-Rich Nuclear Receptor Coactivator 2)-mediated degradation [27,28,29]. SMG6, an endonuclease, interacts with EJC to initiate the degradation by generating cleavage in the proximity of the PTC in the NMD transcript [30,31,32]. SMG5-SMG7 or SMG5-PNRC2 recruits the decapping complex (DCPC) and the deadenylation complex (CCR4-NOT) to remove the cap-binding complex and the poly(A) tail. This subsequently facilitates 5′-to-3′ and 3′-to-5′ RNA degradation, promoted by XRN1 and the RNA exosome, respectively [17,19,20,33]. Recently, it has been revealed that SMG5 can substitute the role of SMG7 to activate NMD, whereas SMG7 requires interaction with SMG5 and phosphorylated UPF1 for complete NMD activity [34,35]. Given that both routes target the same transcripts, SMG5-SMG7 and SMG6-mediated degradation mechanisms are thought to be redundant [36]; they are independent at the same time because downregulation of individual NMD factors (SMG5, 6, or 7) does not inhibit NMD completely [12,37,38].

Besides the EJC-dependent NMD pathway, there exists evidence that shows that the transcripts lacking EJCs are also targeted by NMD. This is called the EJC-independent NMD pathway, which is less well-defined compared to the EJC-dependent NMD pathway. This model (EJC-independent) recognizes unusually long 3′ untranslated regions (3′UTR). This NMD pathway has been demonstrated quite elaborately in yeast and Drosophila, but related NMD mechanisms have also been reported in mammalian cells [39,40,41]. According to this model, the presence of a PTC far upstream of the 3′ end or long 3′UTR hampers the interaction of cytoplasmic polyadenylate-binding protein (PABPC1) to the terminating ribosome. This favors the interaction of ribosome-associated eRF3 to UPF1, rather than to PABPC1, which happens during normal translation termination [42,43].

Although molecular features of NMD targets, as described above, are consistent for many mRNAs, some NMD targets do not follow such rules. Some mRNAs with a PTC can escape NMD, whereas some mRNAs without a PTC can be degraded by NMD. This suggests there must be some exceptions to the canonical NMD regulation, which are responsible for varied NMD efficiency. Several genomic approaches have been applied to predict whether a particular PTC-containing transcript will go for NMD or not. On the basis of cancer genome data analysis, some non-canonical rules have been suggested and validated in some independent experiments [44,45,46]. As discussed, a PTC in a transcript is generated not only through point mutation in the coding region, but also by mutations in the splice site and insertions or deletions (indels), causing a frameshift of the open reading frame. By default, these mutations are considered to result in the loss of function of transcripts with a PTC because of the presumption that these transcripts are bound to be degraded by NMD. However, the genomic data analyses revealed that many of the PTC-containing transcripts, including disease-causing variants, actually evade NMD completely or partially [47,48,49], resulting in the production of truncated proteins. As a predictive estimate, in humans, roughly 50% of the potential PTC variants could partially evade NMD degradation [49]. Transcriptome-wide analyses also identified that many mRNAs encoding full-length proteins are targeted by NMD [8,48,49,50]. Therefore, NMD is a complex process, and there are many poorly understood molecular features that can fine-tune the regulation of the NMD pathway.

## 3. Regulation of NMD in Cancer

**Figure 3:**
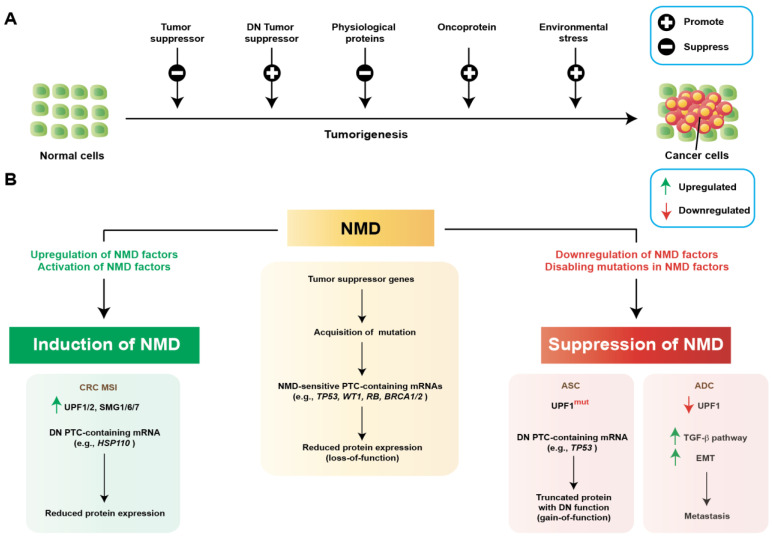
Regulation of NMD in cancer. (**A**) Schematics of different effectors of tumorigenesis, which are targeted by NMD. Tumor cells regulate NMD to target the mRNAs of these effectors to a favorable outcome that can help tumor growth and progression. (**B**) Differential regulation of NMD to promote tumorigenesis. Tumor cells acquire NMD-inducing nonsense mutations preferentially in tumor suppressor genes (e.g., *TP53, WT1, RB, BRCA1/2*) compared to oncogenes. Degradation of tumor suppressors via NMD is favorable for tumor development and growth. Tumor cells often induce NMD by upregulating NMD factors. Induction of NMD favors tumors by degrading proteins, which are toxic or detrimental to tumor cells. For example, in colorectal cancers with microsatellite instability (CRC MSI), NMD was induced by upregulation of several NMD factors (UPF1/2 and SMG1/6/7). Activated NMD helped the degradation of many premature termination codon (PTC)-containing mRNAs that are toxic against CRC MSI, such as a dominant negative (DN) mutant protein HSP110DE9. Tumor cells also often inhibit NMD by downregulating NMD factors or by deactivating mutations (mut) in NMD factors. Suppression of NMD favors tumors by upregulating oncoproteins or activating signaling pathways that favor tumorigenesis, metastasis, or adaptation to environmental stress. For example, disabling mutations in UPF1 in pancreatic adenosquamous carcinoma (ASC) suppressed NMD and upregulated the expression of a truncated *TP53* isoform with dominant negative activity. In human adenocarcinoma (ADC), downregulated UPF1 inhibited NMD activity, which subsequently increased epithelial-mesenchymal transition (EMT) and metastatic events through upregulation of tumor growth factor β pathway (TGF-β). Therefore, tumor cells exploit complex regulation in NMD to favor their growth and progression.

NMD generally protects cells through surveillance and gene expression regulatory mechanisms, as discussed above. However, based on research in recent decades and accumulating evidence, it is now clear that tumor cells often exploit NMD for their survival benefits [51,52]. This is often accompanied either by activation of NMD or suppression of NMD, affecting critical biological processes and subsequently favoring the growth and progression of cancer cells. For example, NMD can facilitate tumor growth by downregulating tumor suppressors, important physiological proteins, or proteins with immune functions (Figure 3). At the same time, suppression of NMD can also facilitate tumor growth, metastasis, or adaptation to environmental stresses by upregulating oncoproteins or activating signaling pathways (Figure 3). Understanding the precise mechanisms of how the NMD pathway regulates such processes is crucial to identifying important cellular vulnerabilities that can be targeted for therapeutic development. Here, we describe several representative examples with critical mechanistic insights linked with tumorigenesis.

### 3.1. Activation of NMD to Promote Tumorigenesis

Tumor cells often leverage NMD mechanisms (both surveillance and regulation of gene expression) to support uncontrolled growth and progression. Considerable evidence indicates that several tumors acquire somatic mutations in tumor suppressor genes, resulting in PTCs in their mRNAs (Figure 3B). These PTC-containing tumor suppressor transcripts are degraded by the NMD, allowing a favorable selection of cancer cells aggravating malignancy. Numerous NMD transcripts have been identified using a gene expression array-based technique, termed gene identification by NMD inhibition (GINI) [50]. Nonsense mutations have been reported in genes that encode well-known tumor suppressors, such as WT1, TP53, RB, and BRCA1/2 [53,54,55,56]. In support of this notion, it was observed that tumor suppressor genes have a higher tendency to acquire nonsense mutations than oncogenes [47].

Tumor cells often leverage induction of NMD to support uncontrolled growth and progression [57,58,59,60,61,62,63,64]. This is often accompanied by upregulated expression of NMD-associated proteins or factors, or activation of NMD factors, through phosphorylation or dephosphorylation or other related mechanisms (Figure 3B). Indeed, TCGA pan-cancer data revealed surprising amplification of several core NMD factors (UPF1, UPF2, UPF3B, SMG1, SMG5, SMG6, and SMG7) in a variety of tumors [57]. In a study investigating colorectal cancer (CRC), an interesting NMD regulation was observed [11]. The study investigated two subgroups: CRC with microsatellite instability (CRC MSI) and CRC with stable microsatellite sequences (CRC MSS). CRC MSI showed higher levels of PTC-containing mRNAs (PTC-mRNAs), due to widespread instability in microsatellite sequences [11]. Interestingly, the authors found upregulated expression of several critical NMD factors in CRC MSI compared to CRC MSS, including UPF1/2 and SMG1/6/7. This upregulation enabled CRC MSI tumors to survive the generation of the higher level of PTC-mRNAs, which were degraded via NMD (Figure 3B). They further showed that experimental suppression of NMD activity upregulated several PTC-mRNAs. Among these were several encoded defective proteins with putative detrimental activity against CRC MSI. One notable example was the HSP110DE9 chaperone mutant with a dominant negative effect against MSI CRC. Inhibition of NMD in vivo impaired tumor growth in CRC MSI, but not in CRC MSS. In a separate situation, if a PTC-containing tumor suppressor gene generates a truncated protein that fully or partially maintains its original function, as opposed to a dominant-negative function, the NMD-mediated degradation of its mRNA may aid in the development of cancer. For instance, patients with germline NMD-sensitive mutations in the tumor suppressor E-cadherin (*CDH1*) gene have a higher tendency to develop hereditary diffuse gastric cancer (HDGC) than people with NMD-insensitive mutations [58]. This is because NMD-insensitive mutations may still be able to produce truncated E-cadherin with normal function [58].

Besides tumor suppressors, other candidates found to be targeted by NMD-inducing mutations include genes encoding proteins involved in RNA metabolism, chromatin remodeling, and DNA repair [61]. Downregulation of these proteins by NMD may compromise important cellular functions, which subsequently creates cellular vulnerability and favors tumorigenesis. One relevant example is stomach adenocarcinoma, where NMD-inducing mutations were identified in three genes involved in RNA metabolism: *EIF5B, LARP4B*, and *PTEN* [62,63,64].

The NMD pathway is often regulated by the signaling pathway. For example, AKT is a serine/threonine kinase (also known as protein kinase B, or shortly, PKB). AKT plays important roles in many cellular functions, such as cell growth and cell cycle progression, genome stability, transcription, protein synthesis, regulation of glucose metabolism, and neovascularization [65,66,67]. Overexpression or activation of AKT is linked to increased cancer cell proliferation [65,66,67]. Misregulation in AKT-regulated pathways is frequently identified in many different cancers, including lung, ovarian, and pancreatic cancers [65,66,67]. It is also commonly misregulated in many other human diseases, such as diabetes, cardiovascular diseases, and neurological defects [65,66,67]. A recent paper unexpectedly discovered a novel role of AKT as a mediator of NMD, which is stimulated by insulin [68]. The authors found that AKT signaling promotes the formation of an alternative EJC that contains CASC3 but is devoid of RNPS1 and UPF2. Furthermore, AKT promotes UPF1 phosphorylation through a distinct mechanism from UPF2, which augments UPF1 helicase activity and is crucial to elicit NMD. It will be interesting to investigate the potential link of the AKT-mediated NMD pathway in tumorigenesis or other human diseases.

### 3.2. Suppression of NMD to Promote Tumorigenesis

Tumor cells often suppress NMD for survival (Figure 3B). For instance, tumor cells often acquire mutations in genes encoding critical factors in the NMD pathway. Some of these mutations compromise the function or expression of the encoded proteins and, subsequently, inhibit NMD. This may elevate the aberrant mRNAs normally degraded by NMD, which can contribute to tumorigenesis. A relevant example in this regard is pancreatic adenosquamous carcinoma (ASC), which is notoriously known for its worse prognosis and aggressive metastatic potential. ASC tumors frequently harbor somatically acquired mutations in *UPF1,* a core component in the NMD pathway [69]. These tumor-specific mutations were shown to generate aberrantly spliced isoforms of UPF1, affecting the essential helicase domain and an important phosphorylation site [69]. The resultant transcripts were predicted to have compromised activity or a dominant negative activity. Indeed, it was found that these tumor-specific mutations in *UPF1* perturbed NMD [69]. This subsequently caused the upregulation of NMD substrate mRNAs, including one notable alternative isoform of p53 mRNA (*TP53*) harboring an in-frame PTC (Figure 3B) [69]. The authors further showed that this alternative isoform of *TP53* encoded a protein with a dominant negative activity and was predicted to contribute to tumorigenesis in ASC. A similar mechanism was also reported in inflammatory myofibroblastic tumors (IMT), where somatic mutations in *UPF1* upregulated an NMD substrate mRNA encoding NF-κB, contributing to immune infiltration associated with IMT [70]. These observations suggest a general protective function of NMD against tumorigenesis. Reduced expression of NMD factors could also suppress NMD. For example, UPF1 is expressed at lower levels in human adenocarcinoma (ADC) compared to normal lungs, which causes downregulated NMD activity in ADC [71]. The study further showed that lower NMD activity promoted the upregulation of several factors in the tumor growth factor β pathway (TGF-β), which subsequently increased epithelial-mesenchymal transition (EMT) and metastatic events (Figure 3B). In another study, it was shown in hepatocellular carcinoma (HCC) that downregulation of UPF1, due to promoter hypermethylation, inhibited NMD and upregulated expression of SMAD7 [10]. Note that SMAD7 is a negative regulator of the TGF-β pathway; therefore, inhibition of NMD dysregulated the TGF-β pathway in this situation [10]. Therefore, suppression of NMD could promote tumorigenesis, either by promoting or inhibiting the TGF-β pathway, based on signaling within the tumor.

## 4. Regulation of Alternative Splicing Coupled to NMD in Cancer

Alternative splicing (AS) is a highly intricate, post-transcriptional mechanism that enables proteome diversity using a limited number of genes. It is estimated that more than 90% of mammalian genes undergo alternative splicing [72]. AS is coordinately regulated by *cis*-elements and *trans*-factors. *Cis*-elements comprise specific sequence motifs in the RNA, also called exonic/intronic splicing enhancers/silencers (ESEs, ISEs, ESSs, and ISSs). In contrast, *trans*-factors are RNA-binding proteins, also called splicing factors (SFs). SFs are often tightly regulated in a tissue-specific manner [73,74]. The formation of functional ribonucleoprotein complexes (RNPs) is coordinated with high precision during splicing to ensure that RNA and cognate proteins are complemented correctly. Impaired biogenesis of RNPs, by disrupting the *cis*-elements or by compromising the RNA-binding activity of SFs, can cause errors in AS, and often produce mRNAs with PTCs. These erroneous mRNAs with PTCs are then recognized and degraded by NMD. This combined action is referred to as AS coupled to NMD (shortly, AS-NMD). The advancement of sequencing technologies in recent decades enabled us to reveal many alternatively spliced mRNA isoforms, which are predicted targets for NMD. As an estimate, in mammals, one-third of the alternative splicing events are non-productive, as they generate a PTC and are degraded by NMD [75]. In recent decades, AS-NMD has been identified as a major driver of tumorigenesis. Many of these tumorigenic AS-NMD events are caused by alterations in splicing factors, such as mutation, abnormal expression, degradation, post-translational modification, etc. Here, we briefly discuss the roles of splicing factors promoting tumorigenic AS-NMD.

### 4.1. Roles of SR Protein Splicing Factors Regulating AS-NMD in Cancer

**Figure 4:**
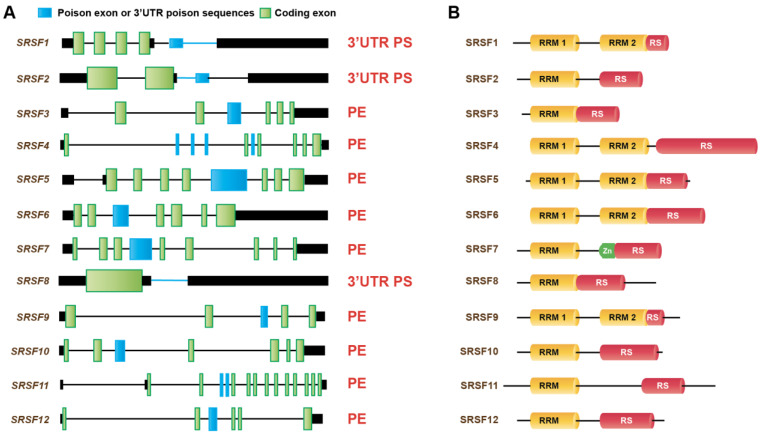
Gene and protein structures of SR protein splicing factors. (**A**) Schematics of SR protein genes with coding exons, non-coding regions, and internal poison exons or 3′UTR poison sequences (not to scale). The presence of poison exons or 3′UTR poison sequences in each gene is shown on the right. (**B**) Schematics of protein structure and domain organization of SR proteins (not to scale). PE: poison exons; PS: poison sequences; RRM: RNA recognition motif; RS: serine-arginine rich domain; Zn: zinc knuckle.

**Figure 5:**
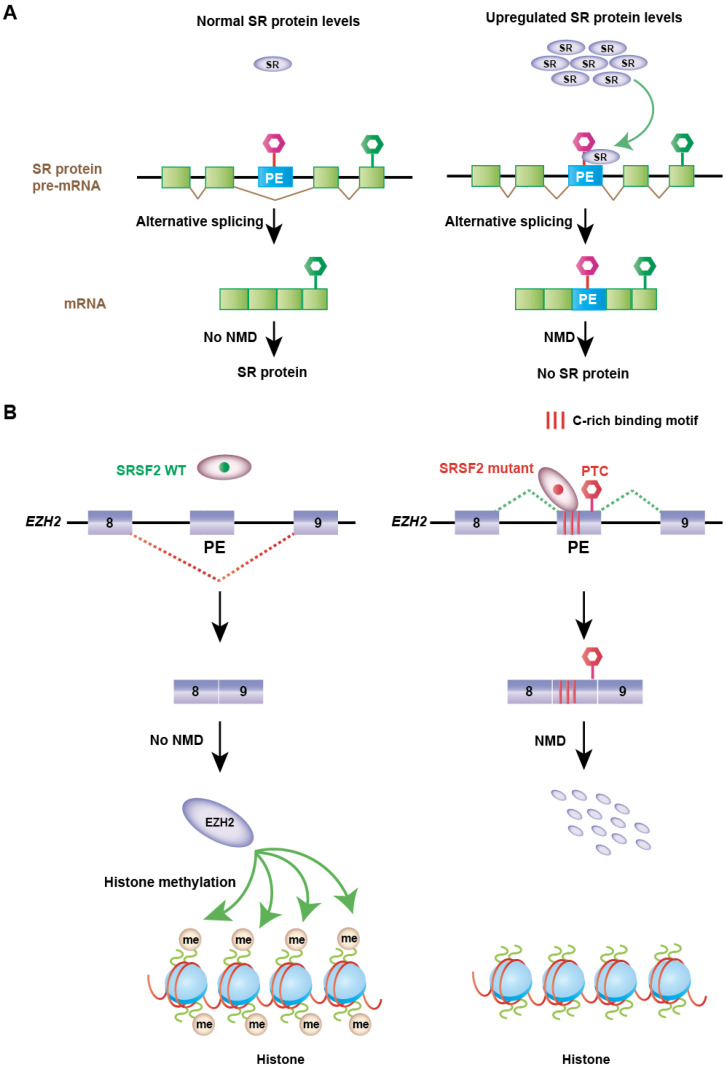
Regulation of AS-NMD in cancer. (**A**) When SR proteins are overexpressed in normal cells, they autoregulate their expression via AS-NMD for homeostasis. SR protein genes harbor ultraconserved regions containing non-coding exons, also called poison exons (PEs) or 3′UTR poison sequences (PSs). When poison exons are included, they introduce a premature termination codon (PTC) and target the mRNAs for degradation. In contrast, splicing of the 3′UTR poison sequences introduces a new exon–exon junction, which marks the original stop codon as a PTC and elicits NMD. SR protein autoregulation via AS-NMD is inhibited in certain tumors. (**B**) SRSF2 is recurrently mutated in hematologic malignancy. One aberrant AS-NMD event promoted by the mutant SRSF2 is the inclusion of a poison exon in *EZH2*. EZH2 catalyzes histone methylation and functions in chromatin remodeling. Mutation in SRSF2 changes its binding preferences for a C-rich motif. This causes the inclusion of the *EZH2* poison exon, which generates a PTC and is degraded by NMD. Therefore, the expression of EZH2 protein is downregulated, contributing to impaired hematopoietic differentiation.

Serine/arginine-rich proteins (SR) belong to a family (SRSF1-SRSF12) of splicing factors and generally function as splicing enhancers (Figure 4) [76]. They contain RNA recognition motifs that bind to RNA and serine/arginine-rich (RS) domains for protein–protein interactions (Figure 4B). When SR proteins are overexpressed in normal cells, they autoregulate their expression via AS-NMD to maintain homeostasis [9,77,78,79,80,81,82,83,84,85]. SR protein genes harbor ultraconserved regions, such as PTC-containing non-coding exons, also called poison exons (PEs), or 3′UTR intronic or poison sequences (PSs) (Figure 4A). The inclusion of poison exons introduces a PTC and targets the mRNAs for degradation by NMD (Figure 5A) [9,77,83]. In contrast, splicing of the 3′UTR intronic or poison sequences introduces a new exon–exon junction, which marks the original stop codon as a PTC and elicits NMD. For example, SRSF2 autoregulates its own expression by promoting the inclusion of a poison exon and retention of an intron in the 3′UTR [78]. This feedback autoregulation is also evident for several other SR proteins, including SRSF1, SRSF3, SRSF4, SRSF5, and SRSF6 [9,77,78,79,80,81,82]. Besides autoregulation, SR proteins also cross-regulate the expression of other SR proteins via AS-NMD [9]. SR proteins are frequently upregulated in a variety of solid tumors [73,74,86,87]. Interestingly, the feedback regulation of SR proteins via AS-NMD was altered in several of these tumors [9]. For example, the Cancer Genome Atlas RNA-sequencing of breast tumors revealed that the inclusions of SR protein poison exons were significantly lower in breast tumors compared to adjacent normal tissues [9]. This suggests that reduced inclusions of poison exons contribute to increased SR protein expression in tumors. Upregulated expression of SR proteins often turns them to function as oncoproteins [73,74,86,87]. For example, slight overexpression of SRSF1 could sufficiently promote the transformation of fibroblasts and mammary epithelial cells [88,89]. Similarly, gastric cancer exhibits increased expression of SRSF7, whereas colorectal cancer exhibits increased expression of SRSF3, SRSF5, and SRSF6 [73,74]. Upregulated SR proteins promote splicing alterations to downstream target genes, some of which function as drivers of oncogenesis. It was reported that overexpression of SRSF1 promotes the skipping of exon 11 of the RON proto-oncogene (*MST1R*) to generate the RONΔ11 isoform, which enhances cell motility and invasion [90]. SRSF1 overexpression also generates an isoform of *BIN1* that lacks tumor-suppressor activity by promoting the inclusion of exon 12a. This isoform with exon 12a lacks the ability to bind to MYC [88].

In addition to regulation in alternative splicing, SR proteins also function in mRNA export, translation regulation, and NMD [73,74,79,86,87,91]. Several SR proteins (SRSF1, SRSF2, SRSF3, SRSF4, SRSF6, and SRSF9) have been shown to enhance NMD [91]. Although SR proteins commonly elicit NMD, the mode of action of individual SR proteins could be different. For example, in the absence of EJC, NMD activity is still observed for SRSF1, but not for SRSF2 [91,92]. This was explained after the observation that SRSF1 increases the binding of NMD factor UPF1 to nuclear-associated mRNAs, bypassing UPF2 recruitment to promote NMD [91]. In contrast, SRSF2-promoted NMD follows the canonical EJC-dependent NMD mechanism, including EJC, UPF3B, UPF2, and UPF1 [92].

As noted earlier, SR proteins are frequently upregulated in a variety of solid tumors. Recurrent mutations in SR proteins are rarely identified, except for SRSF2 in hematologic malignancies, including myelodysplastic syndromes (MDS) and leukemia [73,74,92,93]. Surprisingly, unlike solid tumors, RNA-sequencing data from CD34+ cells of patients with MDS exhibited no significant difference in the inclusion of SR protein poison exons in SRSF2 mutated samples compared to wild-type (WT) samples [9]. These data suggest that solid tumors evade AS-NMD to maintain higher expression of SR proteins, which is not evident in SRSF2-mutated hematological malignancies. Rather, mutations in SRSF2 induce AS-NMD of downstream targeted genes, some of which affect hematopoiesis [92]. One aberrant AS-NMD event promoted by the mutant SRSF2 target is the inclusion of a poison exon in the *EZH2* encoding enhancer of zeste homolog 2 protein. EZH2 catalyzes histone methylation and functions in chromatin remodeling (Figure 5B). Mutation in SRSF2 changes its binding affinity from a G-rich motif to a C-rich motif. This subsequently renders the inclusion of the *EZH2* poison exon in SRSF2-mutated patients [92,93,94]. The resulting transcript generates a PTC and is degraded by NMD. This causes an overall reduction in EZH2 protein levels, which subsequently impairs hematopoietic differentiation. This mechanism was further supported by the observation that restoring EZH2 expression partially rescues hematopoiesis in SRSF2 mutant cells [94]. Another robust aberrant AS-NMD target of mutant SRSF2 is *INTS3* [92,95]. INTS3 is a member of the Integrator complex. This complex plays important functions in transcription initiation, the release of paused RNA polymerase II, small nuclear RNA (snRNA) processing, etc. [95,96]. Altered binding affinity promoted by mutations in SRSF2 causes the retention of two consecutive introns (introns 4 and 5) in *INTS3*. This aberrant regulation generates INTS3 transcript isoforms with a PTC, which are degraded by NMD [92,95].

### 4.2. Roles of Other Splicing Factors and RNA Binding Proteins Regulating AS-NMD in Cancer

Heterogeneous nuclear ribonucleoproteins (hnRNPs) are another group of splicing factors that generally function as splicing repressors [73]. Splicing autoregulation via AS-NMD is also evident in several paralogs of hnRNPs, such as hnRNP L and hnRNP LL, PTBP1 and PTBP2, and hnRNP D and hnRNP DL [83]. When hnRNPs are upregulated, they bind to their own transcripts and promote exon-skipping, leading to the generation of a PTC, and are subsequently degraded by NMD. For example, upregulated PTBP1 (also known as hnRNP I) promotes the skipping of exon 11 of its own transcript, which generates a PTC to control its own expression via AS-NMD [83]. In cancer cells, PTBP1 and PTBP2 promote the skipping of an exon of *SRSF3*, therefore impairing the autoregulation of SRSF3 [83]. This subsequently upregulates SRSF3, which can function as an oncoprotein.

SF3B1 is an essential RNA splicing factor. SF3B1 is frequently mutated in hematologic malignancies, including MDS and leukemia [93]. One aberrant AS-NMD event in SF3B1-mutated patients is the inclusion of a poison exon in the tumor suppressor BRD9 [97]. This is a core component of the non-canonical BAF chromatin-remodeling complex. Mutant SF3B1 recognizes an aberrant branchpoint within BRD9, which promotes the recognition of a poison exon and subsequent degradation of BRD9 mRNA, resulting in the loss of tumor-suppressing activity [97].

RNA-binding proteins (RBPs) are also reported to be important in the development of cancer. For instance, Mex-3 RNA Binding Family Member A (MEX3A) is strongly associated with cancer. It was shown that MEX3A is overexpressed in ovarian cancer tissues and associated with increased proliferation and invasion of ovarian cancer cells [98,99]. Further investigation revealed that TIMELESS is a critical downstream target of MEX3A. TIMELESS promotes the growth and invasion of ovarian tumor cells [98]. Mechanistic analyses revealed that MEX3A promotes the splicing of intron 23 in TIMELESS mRNA, which is critical for the expression of TIMELESS and oncogenic ability [98]. Knockdown of MEX3A causes retention of intron 23 in TIMELESS mRNA and is subsequently degraded by NMD. Therefore, the MEX3A/TIMELESS oncogenic signaling pathway is a key regulator of ovarian cancer, which is suppressed by AS-NMD in normal cells [98]. Another study showed that MEX3A promotes nasopharyngeal carcinoma by positively activating the NF-κB signaling pathway [100]. In inflammatory myofibroblastic tumors, it was demonstrated that somatic mutations in UPF1 triggered alternative splicing of UPF1 [70] and disrupted the NMD pathway. This subsequently caused the upregulation of several NMD targets, including NIK mRNA encoding a potent activator of NF-κB. NIK-dependent NF-κB induction critically contributed to immune infiltration [70].

## 5. NMD under Tumor Microenvironment

The tumor microenvironment during tumorigenesis is characterized by aberrant angiogenesis, hypoxia, acidosis, nutritional deprivation, oxidative stress, and other different cellular stresses. Cancer cells develop adaptability against adverse microenvironments to support growth, proliferation, invasion, and metastasis [101,102]. Interestingly, NMD is often suppressed under a hostile microenvironment. For instance, environmental stress-mediated NMD suppression was promoted by both eIF2α phosphorylation and the mTOR signaling pathway [102]. It was also shown that hypoxia enhanced cellular resistance to the integrated stress response and promoted tumor progression by inhibiting NMD in an eIF2α phosphorylation-dependent manner [101]. The study also suggested that promoting NMD by downregulating the expression of hypoxia-inducible factor 1 (HIF-1) could be an effective therapeutic strategy to target hypoxic tumors.

Nutritional starvation (such as amino acid or glucose) is commonly observed in the tumor microenvironment, due to dysregulated blood vessels and rapid cellular growth. NMD inhibition, induced by amino acid starvation, upregulated the transcripts that enhanced amino acid homeostasis, promoted autophagy, and suppressed genomic noise [7]. A similar study reported that amino acid starvation enhanced the adaptability of yeast by upregulating eIF2α phosphorylation-mediated gene expression [103]. It has been found that cancer cells promote tumorigenesis and enhance resistance against cancer therapy by increasing endogenous antioxidant synthesis under oxidative stress. For instance, SLC7A11, a cystine/glutamate amino acid transport system, was upregulated to enhance cystine transport for the synthesis of glutathione. Similarly, the downregulation of NMD by the tumor microenvironment and cellular stress increased SLC7A11 expression, promoted cystine transport, and enhanced intracellular glutathione synthesis [104]. Therefore, promoting NMD could hamper the adaptability of cancer cells to hostile microenvironments and improve the efficacy of radio- and chemotherapy.

Cancer cells are not tightly regulated by the immune system, unlike microorganisms, because of the lack of strong tumor rejection antigens. Moreover, mRNAs encoding the highly immunogenic neoantigen peptides are often selected for decay by NMD. It was reported that inhibition of NMD suppressed tumor growth by increasing the expression of neoantigen and promoting T cell infiltration in the tumor microenvironment [105]. Pan-cancer data showed that around 30% of frame-shift insertion/deletion mutations in tumor suppressor genes (TSGs) are insensitive to NMD [61]. A similar study confirmed that NMD-escaping mutations exert stronger immune blockade against tumors compared to those with no NMD-escaping mutations [106]. Therefore, stimulation or inhibition of NMD under specific conditions could enhance the efficacy of immune response, as well as immune therapy, against cancer.

## 6. Perspectives and Concluding Remarks

**Figure 6:**
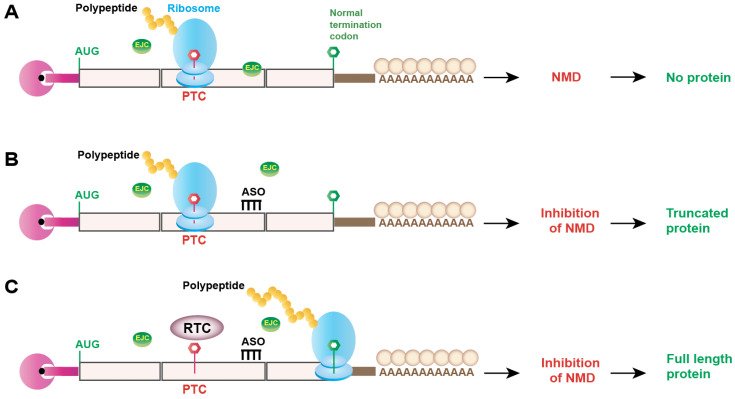
Gene-specific NMD inhibition and targeted augmentation of protein synthesis. (**A**) A premature termination codon (PTC)-containing mRNA is degraded by nonsense-mediated mRNA decay (NMD), inhibiting the expression of the encoded protein. (**B**) Antisense oligonucleotides (ASO) targeting gene-specific exon junction complex (EJC)-binding sites can inhibit NMD, allowing the expression of a truncated protein from a PTC-containing mRNA. The truncated protein may or may not be functional depending on the presence or absence of the functional domain(s). (**C**) Gene-specific ASO-mediated NMD inhibition, along with the readthrough compound (RTC), can allow the expression of a full-length protein from a PTC-containing mRNA.

Studies of NMD in cancer over the last few decades have substantially extended our fundamental understanding of NMD far beyond the physiological regulations in surveillance and transcript abundance. Transcriptome-wide studies of cancer cells revealed that NMD shapes the mutational landscape for selection in favor of tumor survival and globally shapes the transcriptome to support the development and progression of tumor cells [44,45,107]. Although these studies broaden our understanding of the molecular regulations of NMD in cancer, there are certain facts that remain to be explored in the field. Since many of the NMD events are tumor-specific and often fine-tuned in a tissue-specific manner, future studies with comprehensive profiling of NMD targets in different tissues will aid in the understanding of spatiotemporal regulation of NMD in a tumor-specific manner. In the context of tumor evolution, it is imperative to understand which individual step(s) (early, intermediate, or late stage of tumor development) is(are) affected by NMD. This will help to determine the feasibility of targeting NMD at specific stages of cancer therapy. To address the above-mentioned issue, a specific NMD target could be profiled by knockdown and rescue experiments at different stages of tumor development and progression. In the context of molecular regulation, it is also important to know which individual step(s) in the NMD pathway is(are) affected in the journey of a tumorigenic NMD substrate mRNA. This will give the advantage of targeting transcript-specific (or gene-specific) NMD modulation rather than global NMD modulation. Note that global modulation of NMD could show a partial beneficial effect for a particular tumorigenic mRNA substrate, but it will compromise the surveillance regulation of many endogenous mRNAs important for normal physiology and cellular integrity. Therefore, more investigations should be employed for detailed mechanistic characterization of tumor specific NMD target mRNAs. One recent study investigated detailed characterization using a reporter NMD substrate of *HBB* in the context of SRSF2-mutated hematologic malignancies [92]. The authors dissected the metabolism of the NMD substrate in cells expressing SRSF2 WT or SRSF2 mutant. They showed that the SRSF2 mutant promotes stabilization of the exon junction complex (EJC) downstream of the PTC, which subsequently enhances the association of other key NMD factors to elicit mRNA decay. The authors further showed that antisense oligonucleotides (ASO)-mediated targeted blocking of EJC in the NMD substrate mRNA could evade aberrant NMD promoted by mutant SRSF2. This experiment provided an example of mechanism-based targeted inhibition of NMD or AS-NMD in a gene-specific manner (Figure 6). However, restoring the expression of a PTC-containing transcript is predicted to generate a truncated protein (Figure 6). Therefore, this strategy will be beneficial only for those proteins having functional or catalytic activities within the truncated portion of the protein. An alternative strategy could be using ASO in combination with a translation readthrough compound (RTC) that was described by the same group in another study [108]. During translation readthrough in the presence of RTCs, the stop codon is recognized as a triplet coding for an amino acid by the translational machinery, which promotes the translation of a full-length protein from a PTC-containing mRNA [109]. However, NMD limits the efficacy of RTCs due to the degradation of PTC-containing mRNAs [108]. It was shown that ASO-mediated NMD inhibition, along with the readthrough compound G418 targeting PTC-containing *HBB* mRNA, successfully restored the full-length HBB protein with greater efficiency [108]. Therefore, the combinatorial use of ASO and RTC could be a promising targeted therapeutic approach against NMD-associated cancer (Figure 6). For modulating AS-NMD, ASO-mediated splice-switching to generate canonical protein could be an effective therapeutic strategy. In a recent study with an SF3B1-mutated hematologic malignancy, splice-switching ASOs could successfully switch the generation of mRNA from a poison exon-including NMD isoform of BRD9 to the canonical isoform, restoring the expression of BRD9 [97]. They further showed that splice-switching ASOs could reduce tumor size and growth in vivo, suggesting that splice-switching ASOs could be a promising therapeutic strategy to target AS-NMD-associated cancer. Although therapeutic approaches targeting NMD or AS-NMD in several genetic diseases have exhibited clinical success [74,86,87,110], this is still an underexplored area in cancer. This is due to the complex regulation of NMD or/and AS-NMD in cancer because multiple genes are targeted with tumor-specific responses. Elucidating the mechanisms of individual targets will provide valuable information in the development of personalized medicine. Furthermore, the possibility of manipulating several targets simultaneously may provide a more effective strategy to better combat cancer.

## Data Availability

Not applicable.
